# Regulation of cytoskeletal dynamics by redox signaling and oxidative stress: implications for neuronal development and trafficking

**DOI:** 10.3389/fncel.2015.00381

**Published:** 2015-09-30

**Authors:** Carlos Wilson, Christian González-Billault

**Affiliations:** Department of Biology, Faculty of Sciences, Universidad de ChileSantiago, Chile

**Keywords:** redox, cytoskeleton, neurons, development, trafficking

## Abstract

A proper balance between chemical reduction and oxidation (known as redox balance) is essential for normal cellular physiology. Deregulation in the production of oxidative species leads to DNA damage, lipid peroxidation and aberrant post-translational modification of proteins, which in most cases induces injury, cell death and disease. However, physiological concentrations of oxidative species are necessary to support important cell functions, such as chemotaxis, hormone synthesis, immune response, cytoskeletal remodeling, Ca^2+^ homeostasis and others. Recent evidence suggests that redox balance regulates actin and microtubule dynamics in both physiological and pathological contexts. Microtubules and actin microfilaments contain certain amino acid residues that are susceptible to oxidation, which reduces the ability of microtubules to polymerize and causes severing of actin microfilaments in neuronal and non-neuronal cells. In contrast, inhibited production of reactive oxygen species (ROS; e.g., due to NOXs) leads to aberrant actin polymerization, decreases neurite outgrowth and affects the normal development and polarization of neurons. In this review, we summarize emerging evidence suggesting that both general and specific enzymatic sources of redox species exert diverse effects on cytoskeletal dynamics. Considering the intimate relationship between cytoskeletal dynamics and trafficking, we also discuss the potential effects of redox balance on intracellular transport via regulation of the components of the microtubule and actin cytoskeleton as well as cytoskeleton-associated proteins, which may directly impact localization of proteins and vesicles across the soma, dendrites and axon of neurons.

## The Nervous System as a Target for Oxidative Species

The chemical reduction-oxidation (redox) balance commands physiological and pathological responses at different levels ranging from cells to tissues to biological systems. Among organs, the brain is especially vulnerable to oxidation for three main reasons. First, the brain consumes high levels of O_2_—up to 20% of the amount used by the entire body (Sparaco et al., 2006). Given that the brain represents only 2% of the total body mass, metabolites derived from O_2_ in the brain are highly concentrated in a restricted space, increasing the risk of oxidation. Second, Fe^2+^ is abundant in many specific areas of the brain (Gerlach et al., 1994), contributing to non-reversible oxidation. Fe^2+^, the redox-active form of iron, catalyzes the conversion of hydrogen peroxide (H_2_O_2_) into the hydroxyl radical (HO⊕) through the Fenton reaction. The hydroxyl radical represents the most chemically reactive of all reactive oxygen species (ROS; Núñez et al., 2012). Finally, the brain lacks effective mechanisms to remove accumulated pro-oxidative molecules (Halliwell, 1992). Together, these factors necessitate that the brain must utilize effective biochemical measures to counter oxidative stress.

Increased oxidation of molecules in both central and peripheral neurons is often associated with aging, oxidative stress and disease (Andersen, 2004). The term oxidative stress was coined to reflect an imbalance between oxidative and reductive molecules that leads to increased accumulation of pro-oxidant species, with deleterious consequences in most cases (Sies, 1997). This is the case for several neuronal pathologies, including Parkinson’s disease, Alzheimer’s disease, Huntington’s disease and amyotrophic lateral sclerosis (Andersen, 2004). Most sporadic versions of these pathologies are linked to aging and have a documented positive correlation between oxidative stress and development of pathology.

Although oxidation of intracellular components owing to increased oxidative stress is a natural consequence of long-term exposure to pro-oxidant conditions, it is important to remember that physiological synthesis of oxidative species is required for normal cellular function (Rhee, 2006; Dáux and Toledano, 2007). The innate immune response depends on proper synthesis of ROS produced by NADPH oxidases (NOXs). Particularly, phagocytosis, chemotaxis and cellular locomotion of immune cells require basal ROS synthesis (Lambeth, 2004). Other processes requiring physiological concentrations of ROS include thyroid hormone synthesis, Ca^2+^ homeostasis, ion channel dynamics and cytoskeletal remodeling (Bedard and Krause, 2007; Hidalgo and Nunez, 2007; Espinosa et al., 2009; Sakai et al., 2012; Contreras-Ferrat et al., 2014).

In the central nervous system (CNS), enzymatic production of physiological levels of ROS contributes to synaptic plasticity and memory consolidation (Knapp and Klann, 2002; Massaad and Klann, 2011). Genetic models in which gp91^phox^ and p47^phox^ proteins are inactivated (the catalytic and one of the regulatory subunits of the NOX complex, respectively) exhibit abnormal long-term potentiation responses after electrical stimulation, which is an *ex vivo* paradigm to evaluate synaptic plasticity in hippocampal neurons (Kishida et al., 2006). gp91^phox^ and p47^phox^ knockout mice also have decreased consolidation of spatial memory, suggesting a neuronal disorder owing to impaired NOX activity and ROS signaling (Kishida et al., 2006). Humans with chronic granulomatous disease (CGD), an inherited syndrome caused by point mutations in the NOXs proteins gp91^phox^, p47^phox^, p67^phox^ and p22^phox^, affecting the immune response to pathogens, develop cognitive impairments and reduced intellectual coefficients compared with healthy control individuals (Pao et al., 2004). CGD develops during childhood and often requires repeated long-term hospitalization throughout life. It has been proposed that the intellectual deficits in CGD children could be linked to irregular school attendance and that this could even be the main cause for their low intellectual coefficients. However, other infectious diseases with similar periods of hospitalization do not result in cognitive deficits, suggesting that CGD patients develop specific neuronal alterations mainly attributable to reduced NOX activity and decreased ROS synthesis (Pao et al., 2004).

Taken together, these observations suggest that basal physiological ROS synthesis is required for normal cellular function, including the regulation of neurotransmission, but that high and unregulated ROS concentrations lead to oxidative stress and disease.

## General Overview of Redox Balance

### Intracellular Sources of ROS

The main oxidative species derived from O_2_ are H_2_O_2_, HO⊕ and the superoxide anion (O_2_⊕^–^) and are collectively called ROS (Bedard and Krause, 2007). Nitrogen (N_2_), the principal gas in the atmosphere we breathe, also induces intracellular oxidation via the production of physiologically reactive nitrogen species (Weidinger and Kozlov, 2015).

The main sources of intracellular ROS are mitochondria and NOXs. In mitochondria, complexes I and III of the electron transfer chain produce the short-lived O_2_⊕^–^, a radical derived from O_2_ (Murphy, 2009; Bigarella et al., 2014). No intracellular signaling pathway that regulates mitochondrial superoxide synthesis has yet been described, suggesting that mitochondria could be a source of constitutive ROS production. Synthesis and release of ROS from mitochondria depend on the tissue and its intrinsic metabolism. Mitochondrial dysfunction quickly leads to oxidative stress that targets DNA, membrane lipids and proteins, directly affecting cell physiology (Tahara et al., 2009).

NOXs represent the other major cellular source of ROS (Bedard and Krause, 2007). The NOX family includes seven members that catalyze the production of O_2_⊕^–^ in an NADPH-dependent reaction. The family is composed of five canonical NOXs (NOX1 to NOX5) and two dual oxidases (Duox1 and Duox2; Lambeth et al., 2007). NOXs represent the main enzymatic source of ROS, and several signal transduction pathways are involved in their regulation (Dang et al., 2001; Park et al., 2001; Chen et al., 2003; Hoyal et al., 2003). NOX1, NOX2 and NOX4 are expressed in the CNS (Sorce and Krause, 2009), with NOX2 being the principal enzyme expressed in neurons. NOX2 can produce superoxide by itself but requires interaction with regulatory proteins for stabilization and to increase ROS levels under physiological circumstances. Together with its partners p22^phox^, p47^phox^, p67^phox^ and p40^phox^, NOX2 synthesizes superoxide to meet the physiological requirements of neurons (Bokoch and Diebold, 2002; Glogauer et al., 2003; Nauseef, 2004; Decoursey and Ligeti, 2005).

### ROS as Signaling Molecules

Superoxide reactivity is fairly low, mainly owing to its short life-time and restricted diffusion area (Weidinger and Kozlov, 2015). However, superoxide can be converted to H_2_O_2_ either spontaneously or enzymatically via superoxide dismutase (Núñez et al., 2012). H_2_O_2_, the most stable ROS, is converted to H_2_O by several antioxidant enzymes, e.g., glutathione peroxidase and catalase, and this is likely the reason why oxidative modifications induced by H_2_O_2_ are transient and reversible (Weidinger and Kozlov, 2015). Thus, under normal conditions, the synthesis of superoxide and H_2_O_2_ are enzymatically regulated and their levels remain under a physiological threshold. In the presence of Fe^2+^, however, H_2_O_2_ is rapidly converted to HO⊕ through the Fenton reaction (Núñez et al., 2012). Hydroxyl radicals modify molecules in a non-reversible way, leading to permanent modifications of proteins and other targets. To consider ROS as signaling molecules, they should meet certain spatial and regulatory criteria, namely they should be produced locally and their levels regulated by intracellular molecular systems. According to these criteria, superoxide and H_2_O_2_, but not HO⊕, are considered signaling molecules (Dáux and Toledano, 2007; Janssen-Heininger et al., 2008; Gerich et al., 2009).

### Regulation of Protein Function by Oxidation via Post-Translational Modification

Cysteine thiol groups (SH) of proteins are the main targets for oxidation (Stadtman and Berlett, 1997). In general terms, oxidation of SH groups leads to glutathionylation, nitrosylation and disulfide bond formation. These modifications are enzymatically reversed through the glutaredoxin (Grx), thioredoxin and peroxiredoxin systems, among others (Ghezzi, 2005; Shelton et al., 2005; Janssen-Heininger et al., 2008). Oxidation also leads to sulfenic acid formation and protein carbonylation, two non-reversible modifications that permanently affect protein structure and function (Bigarella et al., 2014).

The functions of several proteins, including cytoskeletal proteins, depend on ROS signaling and oxidation (Sparaco et al., 2006). Several studies have shown that redox balance affects both *in vitro* and *in vivo* cytoskeletal dynamics, which directly impacts cell morphology and morphometrics. In neurons, cytoskeletal rearrangement commands cell development, polarization and neurotransmission (Jaworski et al., 2009; Hoogenraad and Akhmanova, 2010; Stiess and Bradke, 2011; Caceres et al., 2012; Gonzalez-Billault et al., 2012). Neurons are highly polarized cells, having a cell body from which emerge several dendrites and an axon to establish functional communication networks with other neurons and glial cells (Caceres et al., 2012). Acquisition of this morphology depends directly on the dynamics of actin microfilaments and microtubules (Neukirchen and Bradke, 2011). Given the influence of the redox state on neuronal function and its potential role in modifying the cytoskeleton, it is interesting to review the contribution of the redox balance to cytoskeletal organization in neurons.

## Contribution of Redox Balance to Organization of the Neuronal Cytoskeleton

### Redox State of Neuronal Cytoskeleton Proteins

The main components of the neuronal cytoskeleton network, namely actin microfilaments, microtubules and neurofilaments, are susceptible to oxidation (Sparaco et al., 2006). Post-mortem histological studies from non-pathological human samples have revealed a basal pool of glutathionylated proteins in the prefrontal cortex, cerebellum and spinal cord. Cellular analysis of the prefrontal cortex revealed that neurons are more highly glutathionylated than oligodendrocytes and astrocytes. Biochemical analysis revealed that actin, tubulin and neurofilaments are glutathionylated, suggesting that the redox state of neurons and cytoskeletal proteins under basal conditions is slanted toward oxidation (Sparaco et al., 2006). The relevance of cytoskeleton oxidation depends on the spatiotemporal context in which a defined modification occurs as well as the source of the ROS. Whereas physiological ROS production is needed for proper cytoskeleton polymerization, oxidation tends to disrupt polymerization and impair cytoskeletal dynamics under oxidative stress conditions (Munnamalai and Suter, 2009; Hung et al., 2010; Morinaka et al., 2011; Wilson et al., 2015).

### Actin Modification and Regulation of F-Actin Dynamics by Oxidative Species

**Figure 1:**
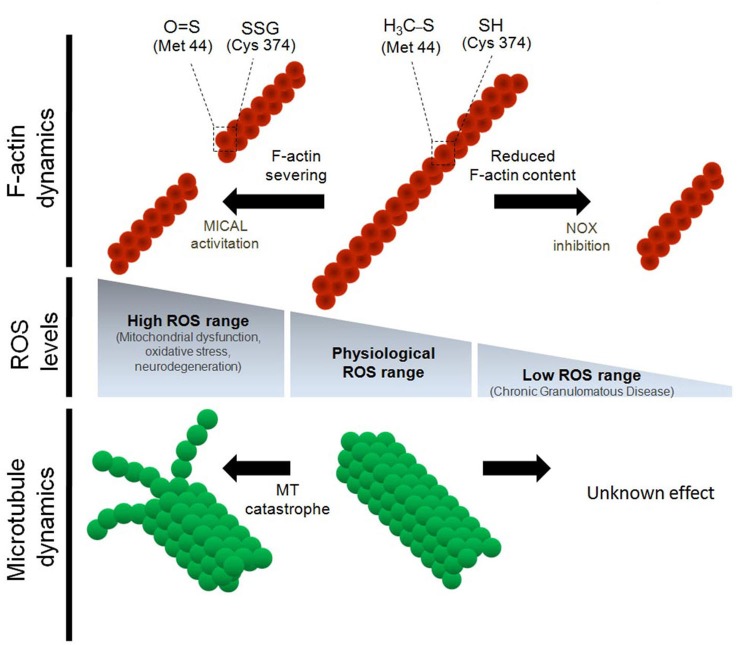
**Regulation of the cytoskeleton dynamics by differential reactive oxygen species (ROS) levels.** Physiological concentrations of ROS are needed for proper F-actin and microtubule dynamics both *in vitro* and *in vivo*. Oxidative stress induces the severing of F-actin and impairs microtubule polymerization. In the particular case of actin monomers, both Cys 374 and Met 42 residues are targets for oxidative molecules during oxidative stress conditions. Oxidation of Cys 374 leads to actin glutathionylation. On the other hand, oxidation of Met 44, which occurs specifically by MICAL1, leads to Met oxidation and severing of the actin filaments (Hung et al., 2011). Despite both α and β tubulin have several Cys residues that are susceptible to oxidation, it has not been described which Cys residues of tubulin monomers are modified by oxidation. In contrast, down-regulation of ROS synthesis, mainly through NOX inhibition, decreases the F-actin content and alters the dynamic properties of the actin cytoskeleton. None of the post-translational modification of actin monomers have been described after NOX inhibition and ROS down-regulation. Finally, the effect of the inhibition of ROS synthesis (below physiological levels) on microtubule dynamics has not been explored.

Intracellular ROS production is needed for proper cell migration and chemotaxis, which are actin-dependent processes (Roberts et al., 1999; Ambruso et al., 2000; Kim and Dinauer, 2001). Actin monomers contain 5 Cys and 16 Met residues (Dalle-Donne et al., 2002, 2003). Of these, only Cys 374 is fully exposed to the cytoplasm (Dalle-Donne et al., 2003). By contrast, Met 44, 47 and 355 are prominently exposed to the cytoplasm and Met 176, 190, 227 and 260 are also susceptible to the action of oxidative molecules (Dalle-Donne et al., 2002). *In vitro* assays suggested that an oxidative environment inhibits actin polymerization (Dalle-Donne et al., 2003). In addition, both Cys and Met residues can be carbonylated after *in vitro* treatment with hypochlorous acid (HOCl), a common derivative product released by leukocytes during the initial phase of the immune response. This polymerization assay was designed to recapitulate *in vitro* the exact oxidative environment of the immune cell response; importantly, this environment inhibits actin polymerization (Dalle-Donne et al., 2001). This analysis also revealed that specific glutathionylation at Cys 374 decreases actin polymerization (Dalle-Donne et al., 2003). In neutrophils, glutaredoxin 1 (Grx1), a deglutathionylating enzyme that reduces oxidized Cys residues, is needed to maintain actin dynamics (Sakai et al., 2012). ROS depletion using the NOX inhibitor diphenyleneiodonium or Grx1 overexpression decreases actin glutathionylation, increases the amount of filamentous actin (F-actin) and impairs proper cellular migration. In contrast, loss of Grx1 function via knockdown and knockout strategies increases the amount of glutathionylated actin and decreases the level of F-actin. Sakai et al. (2012) proposed that physiological levels of ROS and redox balance regulate actin dynamics, which are required for chemotaxis and migration of immune cells. On the other hand, physiological ROS levels induce local membrane protrusions in marsupial kidney epithelium Ptk1 cells in a mechanism that involves cofilin, the actin-related protein 2/3 complex (Arp2/3) and the extracellular signal–regulated kinase (ERK), thereby enhancing the retrograde flow of actin at the leading edge of these migrating cells (Taulet et al., 2012). Figure 1 summarizes the effect of the oxidative power on actin polymerization and F-actin dynamics.

ROS also target the actin cytoskeleton in neuronal cells. The Semaphorin/Plexin signaling pathway represents a major repulsive cue for axonal guidance (Hung and Terman, 2011). Semaphorin 3A (Sema3A) signaling induces a local increase in H_2_O_2_ at the growth cones of dorsal root ganglion neurons by activating MICAL1 and MICAL3 (Morinaka et al., 2011). MICAL1 is a binding partner for the cytosolic domain of Plexin A, a Sema3A receptor (Terman et al., 2002). The principal target of MICAL1 is actin (Hung et al., 2010, 2011; Giridharan and Caplan, 2014). In bristle cells of Drosophila melanogaster, oxidation of actin at Met 44 by MICAL1 severs actin filaments (Hung et al., 2011). MICAL1-dependent actin oxidation is reversed by Selenoprotein R (SelR) activation, restoring F-actin dynamics and polymerization (Hung et al., 2013). After neuronal injury, Sema3A levels rise above normal levels to inhibit the ability of axons to regenerate and regrow. These findings represent a redox mechanism by which Sema3A induces the collapse of axonal growth cones and impedes axon guidance, supporting the hypothesis that redox imbalance—particularly oxidative stress—leads to neuronal damage. Therefore, MICAL1 contributes to axonal pathfinding by regulating ROS signaling at dorsal root ganglion growth cones. In addition to directly regulating the actin redox balance, MICAL1 may modify actin dynamics by promoting interaction between proteins. MICAL1 interacts with Cas and CasL proteins, which may be involved in cross-talk between actin and intermediate filaments (Suzuki et al., 2002). Moreover, MICAL1 negatively regulates the nuclear dbf2-related kinase NDR2 in non-neuronal cells (Zhou et al., 2011). NDR1 and NDR2 contribute to targeting the Par3/Par6/aPKC complex to growing axons, a mechanism that promotes neuronal polarity (Yang et al., 2014). Together, these findings suggest that MICAL1 both directly and indirectly regulates the neuronal actin cytoskeleton, contributing to neuronal function and development under both normal and stress conditions.

**Figure 2:**
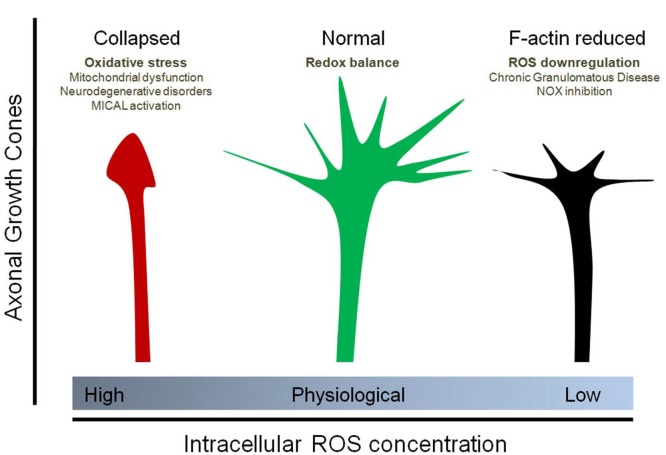
**Effect of physiological and non-physiological ROS on axonal growth cone organization.** Oxidative stress (due to mitochondrial dysfunction, Sema3A/Plexin signaling and MICAL1 activation, neurodegenerative disorders, among others) induces the collapse of the axonal growth cones of neurons. In contrast, down-regulation of ROS synthesis, particularly due to NOX inhibition as well as Chronic Granulomatous Disease (CGD), decreases the F-actin content at the growth cone and affect filopodial dynamics by decreasing the number and length of filopodia at the axonal growth cone. Physiological concentrations of ROS, principally maintained by NOX enzymes and basal mitochondrial activity, are needed for a proper organization of the growth cones. In this context, the implicances for vesicular trafficking due to altered cytoskeleton dynamics has not been explored.

In the marine mollusk Aplysia, NOX-derived ROS are required to maintain proper F-actin dynamics in the growth cones of bag cells (Munnamalai and Suter, 2009; Munnamalai et al., 2014). Inhibition of NOX activity using pharmacological inhibitors like apocynin, diphenyleneiodonium and VAS2870 reduce F-actin content in these growth cones and reduce both retrograde actin flow and neurite outgrowth, supporting the idea that actin dynamics and neurite elongation require basal NOX activity (Munnamalai and Suter, 2009). Cultured embryonic hippocampal neurons that express the mutant P156Q p22^phox^, which down-regulates ROS synthesis by the NOX complex, show a decrease in the number, length and lifetime of filopodia at axonal growth cones (Wilson et al., 2015). Moreover, lamellar actin organization of stage 1 neurons, the initial morphology from which neurons develop, is disrupted after loss of NOX function (Wilson et al., 2015). Together, this evidence supports the hypothesis that local ROS signaling is needed to maintain normal F-actin dynamics in neurons and is consistent with other reports proposing that ROS are needed to support membrane protrusions, lamellar structures and filopodia at the leading edge of migrating cells (Taulet et al., 2012). Interestingly, NOX2 and p40^phox^co-distribute with F-actin at the growth cone of neuronal bag cells in Aplysia, suggesting that local ROS production may be involved in neurite outgrowth (Munnamalai et al., 2014). NOXs are expressed in axons and dendrites of embryonic and adult neurons, suggesting that local ROS synthesis that may be involved in filopodial dynamics and neurite growth (Tejada-Simon et al., 2005; Wilson et al., 2015). Figure 2 summarizes the differential effect of ROS on the organization of the growth cones of axons.

### Tubulin Modification and Regulation of Microtubule Dynamics by Oxidative Species

α-Tubulin and β-tubulin contain 12 and 8 Cys residues, respectively, and each of these residues can be oxidized by endogenous and exogenous oxidizing agents (Luduena and Roach, 1991; Löwe et al., 2001; Landino et al., 2002, 2004a). The functions of these Cys residues are linked to GTP binding, microtubule polymerization and drug response (Mellon and Rebhun, 1976; Luduena et al., 1985; Luduena and Roach, 1991). In *in vitro* polymerization assays using purified tubulin from adult bovine brain, oxidative species added to the reaction medium dramatically reduced tubulin polymerization. Peroxynitrite (ONOO^–^), a ROS produced from the reaction between superoxide and nitric oxide (NO⊕), progressively oxidizes the thiol groups of tubulin monomers, thereby decreasing the ability of microtubules to polymerize *in vitro* (Landino et al., 2007). The same results were obtained with NO⊕ and nitroxyl donors. Moreover, ONOO^–^ promotes disulfide bond formation between α- and β-tubulin (Landino et al., 2004a). In addition, *in vitro* assays revealed that tubulin is glutathionylated after treatment with ONOO^–^, and that this modification is reversed by the glutathione/glutathione reductase system, composed of glutathione, glutathione reductase, Grx and NADPH (Landino et al., 2004a). The reversal of tubulin glutathionylation by this system is interesting because intracellular signaling pathways may modulate microtubule polymerization in a reversible manner. Figure 1 summarizes the effect of high oxidative power on microtubule dynamics. However, the inhibition of ROS synthesis below a physiological range has not been explored in terms of tubulin modifications nor microtubule dynamics.

Another layer of regulation is provided by proteins that stabilize or destabilize microtubules. Microtubule-associated protein 2 (MAP2) and tau are MAPS that specifically regulate MT polymerization in dendrites and axon. MAP2 and tau contain one and seven Cys residues, respectively (Lewis et al., 1988). Oxidation of MAP2 and tau Cys residues decreases microtubule polymerization *in vitro*, suggesting that redox balance regulates tubulin not only through direct interaction but also by regulating their stabilization by MAPs (Landino et al., 2004b). It is plausible that oxidized/reduced MAPs present differential microtubule stabilization. Moreover, binding of MAPs to microtubules may promote differential regulation of molecular motors in axons and dendrites (Dixit et al., 2008), affecting trafficking and cargo destination. Therefore, redox-dependent MAP modifications may be an additional mechanism for regulating cytoskeletal dynamics in neurons. Indeed, increased nitrosylation of MAP1B at Cys 2457 is involved in neurite retraction through a mechanism that couples microtubule stability and dynein function (Stroissnigg et al., 2007; Villarroel-Campos and Gonzalez-Billault, 2014).

Microtubule function depends on its intrinsic polymerization properties (Mitchison and Kirschner, 1984, 1988) as well as the specific tubulin isotype (Kavallaris, 2010) and post-translational modifications (Janke, 2014). Microtubule proteins can be modified by redox state, but understanding the functional consequences of such modifications can be challenging. For example, tubulin modifications induced by ONOO^–^ treatment *in vitro* can be difficult to interpret because ONOO^–^is unstable at physiological pH, and thus *in vitro* microtubule polymerization assays are performed at basic pH (typically pH 10). In addition, tubulin is glutathionylated in both cell-specific and tissue-specific ways (Sparaco et al., 2006, 2009). Prefrontal cortex, cerebellum and spinal cord tissue samples from non-pathological humans exhibit tubulin glutathionylation under basal conditions. In addition, after treatment with oxidized glutathione, neurons are preferentially glutathionylated compared with astrocytes and oligodendrocytes (Sparaco et al., 2006). Thus, it seems that tubulin Cys residues can be modified by redox balance in both *in vitro* and *in vivo* contexts.

Neurite outgrowth is affected by tubulin oxidation in cellular models. In the motor neuron-derived neuroblastoma cell line NSC34, oxidation induced by oxidized glutathione promote the formation of retraction bulbs and thin axon-like processes (Carletti et al., 2011). Under these conditions, glutathionylated tubulin levels are increased and interestingly, tyrosination of α-tubulin is simultaneously decreased. These findings suggest that an oxidative cytoplasmic environment induces tubulin glutathionylation, leading to neurite retraction and degeneration. Moreover, in Friedrich’s ataxia, a neuropathological condition characterized by degeneration of spinal cord pathways, immunohistochemical studies in motor neurons revealed co-distribution of tyrosinated tubulin and glutathionylated proteins (Sparaco et al., 2009). In the future, it will be necessary to establish whether there is a causal relationship between tubulin glutathionylation and changes in microtubule dynamics.

Studies in cultured primary neurons exploring phenotypes and mechanisms underlying the regulation of microtubule dynamics by redox state are still preliminary. However, there is some indirect evidence suggesting a putative link between microtubule polymerization and ROS balance. CRMP-2, a molecular regulator of microtubule polymerization, is oxidized at Cys 504, inducing homodimerization. These dimers can form a transient complex with thioredoxin, which creates a docking site for the protein kinase GSK3-β. GSK3-β-dependent CRMP-2 phosphorylation is linked to growth cone collapse in cultured dorsal root ganglion cells, recapitulating some molecular pathways involved in the initial steps of neurodegeneration (Morinaka et al., 2011). Therefore, new research may help establish a direct link between regulation of microtubule dynamics and redox balance in neuronal systems.

## Participation of Oxidative Species in the Central Nervous System and Neuronal Development

Genetic models that reduce ROS production in the nervous system, such as gp91^phox^ and p47^phox^ knockout mice, are characterized by a macroscopically normal brain overall, including the cerebral cortex and hippocampus (Kishida et al., 2006). However, they display cognitive impairments as well as impaired synaptic plasticity, a phenomenon that involves, among other molecular events, cytoskeletal remodeling (Kishida et al., 2006). Therefore, a complete understanding of the contribution of ROS to normal nervous system physiology is important. Some insights have emerged in the last few years. For example, ROS levels have been shown to contribute to the commitment of neuronal progenitors to differentiate into mature neurons (Forsberg et al., 2013; Quadrato and Di Giovanni, 2013; Forsberg and Di Giovanni, 2014). Along the same line, loss of NOX function (using either neurons from NOX2 knockout mice or NOX2 knockdown in cultured neurons) has been shown to decrease the proliferation rate and number of neural stem cells *in vivo* and *in vitro*, suggesting that physiological ROS levels derived from NOX2 are needed to maintain a basal population of adult hippocampal neuronal progenitors (Dickinson et al., 2011). It was recently observed that inhibition of NOX functions alter normal neuronal polarization and reduces axonal growth (Wilson et al., 2015). Similarly, differentiation of cerebellar granule neurons involves glutathione homeostasis and NOX activity (Olguín-Albuerne and Morán, 2015). Of note, MICAL1 knockout mice exhibit abnormal mossy fiber lamination, aberrant F-actin content and decreased Rab6 trafficking to the growth cones of hippocampal neurons, suggesting a role in the development of mossy fiber axons and of specific sub-areas of the hippocampus and supporting the notion that redox balance is needed for development of brain tissues (Van Battum et al., 2014). Together, these lines of evidence support a new hypothesis in the field of ROS, that the physiological and controlled production of ROS is needed for signaling and development in neurons. In the future, it will be important to address *in vivo* functions for ROS from various sources and their involvement in neuronal differentiation, migration and axonal guidance.

A major challenge in this field is to understand the specificity that redox imbalance have on cytoskeleton proteins compared to DNA or lipids. This is especially important considering that modifications on these molecules could also affect neuronal functions and morphology. In fact, ROS contributes to the transcription of several genes associated with metabolism, cell cycle and development (Bigarella et al., 2014), supporting the notion that ROS have pleiotropic effects on sub-cellular components. Moreover, there are technical concerns about the quantification of ROS in cells. Both ROS and cytoskeleton proteins are dynamic cellular elements with short life-times. Thus, it is hard to establish a correlation between local synthesis of ROS and the oxidation of actin filaments and microtubules in a living cell. New genetic tools based on fluorescence microscopy have emerged in the last years to measure ROS levels (Mishin et al., 2015). Genetically encoded probes to measure ROS combined with cytoskeleton biosensors will be necessary to define the spatial and temporal association between ROS synthesis and cytoskeleton remodeling.

## Emerging Concepts in the Contribution of Redox Balance to Vesicle Trafficking

Intracellular trafficking is highly dependent on actin microfilaments, microtubules and molecular motors such as myosin, kinesin and dynein. In addition, members of the Rab family of small GTPases are essential for targeting components to discrete domains within cells. Several Rab proteins derived from the trans-Golgi network, the early/late endosome and recycling endosomes regulate neurite outgrowth and development (Villarroel-Campos et al., 2014). In preceding sections, we discussed how changes in ROS content may target tubulin and actin dynamics to regulate the tracks for intracellular trafficking. However, the link between redox balance and the vesicle components involved in neuronal trafficking remains poorly understood.

Several studies correlate MICAL activity with trafficking. It has been recovered as an interacting partner for several members of the Rab family in yeast two-hybrid experiments (Fukuda et al., 2008). MICAL1 deletion leads to aberrant destination of the IgCAM cell adhesion molecules to the growth cones of cultured hippocampal neurons in a Rab6- and actin-dependent mechanism, establishing a link between redox state and vesicle trafficking in neurons (Van Battum et al., 2014). Interestingly, MICAL1 interacts with Rab1, which is involved in vesicle trafficking from the endoplasmic reticulum to the Golgi (Fischer et al., 2005). Additionally, MICAL3 interacts with Rab8, which in turns interacts with Rab6 to promote exocytosis of secretory vesicles (Van Battum et al., 2014). Moreover, expression of a mutant isoform of MICAL3 in HeLa cells induces accumulation of vesicles at the cell cortex by inhibiting vesicle docking with the plasma membrane, ultimately decreasing release of vesicle contents (Grigoriev et al., 2011). This suggests a link between Rab-dependent vesicle trafficking and ROS. Zinc deficiency has been shown to decrease tubulin polymerization via oxidation of tubulin thiol groups (Mackenzie et al., 2011). Interestingly, tubulin oxidation also impairs translocation of the transcription factor NFκB to the nucleus, suggesting a link between redox state and microtubule-dependent trafficking in a cellular model (Mackenzie et al., 2011).

In addition to ROS, NO also plays a role in terms of neuronal function and vesicle trafficking. A recent report suggest that NO reduces the expression of the molecular motors KIF5 and KIF21B and it decreases the length of the axons of cultured cortical neurons (Redondo et al., 2015). Authors hypothesize that NO exposure could affect KIF-dependent vesicle trafficking required for normal axonal growth. In fact, axonal retraction and NO release are key issues in some neurodegenerative disorders, like Parkinson’s disease (More et al., 2013; Tripathy et al., 2015). However, authors did not explored vesicle movement after NO exposure and this issue does not allow to conclude a direct effect of NO on axonal trafficking through cytoskeleton regulation. Moreover, G-protein coupled receptors that respond to NO (NO/CG) contributes positively to the physiology of neurons and neurotransmission (Hardingham et al., 2013; Russwurm et al., 2013). In summary, oxidative molecules signaling is an emerging concept in the field of cytoskeleton regulation and further studies will be required to understand the contribution to the vesicle trafficking and its impact on the physiology of neurons.

## Concluding Remarks and Future Perspectives

ROS influence many different cellular functions under both physiological and pathological conditions. The targets of ROS include DNA, lipids and proteins. Among these, cytoskeletal proteins can be modified *in vitro* and *in vivo* by redox molecules. An imbalance between oxidative and reductive species leads to oxidative stress, which affects the polymerization of both F-actin and microtubules. In contrast, down-regulation of ROS also affects normal cytoskeletal organization, impacting the morphology, development and physiology of cells and neurons. Neuronal development and specification of neuronal compartments depend on redox homeostasis, in a mechanism that involves regulation of the actin cytoskeleton by the NOX complex. Further exploration of the role of redox balance in regulating microtubule dynamics in cellular models is required. Abnormal polymerization of actin microfilaments and microtubules directly affects vesicle trafficking and specific cargo delivery throughout the soma, dendrites and axon. However, the regulation of vesicle trafficking and protein sorting by redox balance represents an unexplored field despite strong evidence in several cellular contexts that cytoskeletal proteins are targets of oxidative species. Moreover, the contribution of redox balance to the interaction between the cytoskeleton and cytoskeleton-associated proteins such as myosins, dyneins and kinesin molecular motors has not been studied, and such analysis may reveal direct effects on vesicle trafficking and cargo destination. New evidence has emerged concerning the dissection of the cellular sources of ROS that can modulate cytoskeletal dynamics. The development of new ratiometric microscopy tools to characterize the spatiotemporal production of ROS may give other important clues about how redox balance controls neuronal physiology.

## Funding

This work was funded by CONICYT doctoral fellowship 21120221 to CW and by grants ACT-1114 and Fondecyt 1140325 to CG-B.

## Conflict of Interest Statement

The authors declare that the research was conducted in the absence of any commercial or financial relationships that could be construed as a potential conflict of interest.
